# Numerical simulation of closure performance for neo-aortic valve for arterial switch operation

**DOI:** 10.1186/s12938-016-0264-0

**Published:** 2016-12-28

**Authors:** Zhaoyong Gu, Youlian Pan, Aike Qiao, Xingjian Hu, Nianguo Dong, Xiaofeng Li, Yinglong Liu, Deguang Shang

**Affiliations:** 1 College of Life Science and Bio-Engineering, Beijing University of Technology, Pinleyuan, Chaoyang District, Beijing, China; 2 College of Mechanical Engineering and Applied Electronics Technology, Beijing University of Technology, Pinleyuan, Chaoyang District, Beijing, China; 3 Department of Cardiovascular Surgery, Union Hospital, Tongji Medical College, Huazhong University of Science and Technology, Jiefang Avenue, Qiaokou District, Wuhan, China; 4 Center of Infant Heart, Beijing Anzhen Hospital, Capital Medical University, AnZhen Road, Chaoyang District, Beijing, China

**Keywords:** Arterial switch surgical planning, Structural finite element model, Neo-aortic root

## Abstract

**Background:**

Modeling neo-aortic valve for arterial switch surgical planning to simulate the neo-aortic valve closure performance.

**Methods:**

We created five geometrical models of neo-aortic valve, namely model A, model B, model C, model D and model E with different size of sinotubular junction or sinus. The nodes at the ends of aorta and left ventricle duct fixed all the degrees of freedom. Transvalvular pressure of normal diastolic blood pressure of 54 mmHg was applied on the neo-aortic valve cusps. The neo-aortic valve closure performance was investigated by the parameters, such as stress of neo-aortic root, variation of neo-aortic valve ring as well as aortic valve cusps contact force in the cardiac diastole.

**Results:**

The maximum stress of the five neo-aortic valves were 96.29, 98.34, 96.28, 98.26, and 90.60 kPa, respectively. Compared among five neo-aortic valve, aortic valve cusps contact forces were changed by 43.33, −10.00% enlarging or narrowing the sinotubular junction by 20% respectively based on the reference model A. The cusps contact forces were changed by 6.67, −23.33% with sinus diameter varying 1.2 times and 0.8 times respectively.

**Conclusions:**

Comparing with stress of healthy adult subjects, the neo-aortic valve of infant creates lower stress. It is evident that enlarging or narrowing the sinotubular junction within a range of 20% can increase or decrease the maximum stress and aortic valve cusps contact force of neo-aortic valve.

## Background

The arterial switch operation (ASO) is now preferred surgical approach to treat complete transposition of the great arteries (TGA) presenting in the neonatal period [1]. Although this surgery is thought to be an improvement compared with the earlier procedures, late cardiac complications have been reported in children, including pulmonary artery stenosis, neo-aortic valve insufficiency, and coronary obstruction [1–3]. Neo-aortic valve insufficiencies are approximate 15% after a 75 month follow-up [4]. At least moderate neo-aortic regurgitation is present in 3.4% [5].

Arterial switch operation involves four steps generally, such as closure of intracardiac defects, coronary transfer, aortic reconstruction and pulmonary artery reconstruction [6]. For coronary transfer, the dimension of excised aortic wall can control the sinus diameter (SD). Additionally, for aortic reconstruction, the diameter of pulmonary artery is larger than that of aorta on aortic anastomosis site. The surgical technique includes pulmonary artery constriction or patch enlargement of ascending aorta [7–9]. How to choose the size of STJ and SD of neo-aortic valve during the intraoperative period and profitable is still an open problem.

A structural model of neo-aortic valve for ASO was developed for finite element analysis so as to simulate closure performance of neo-aortic valve with the different size of sinotubular junction (DSTJ) and SD. Different geometric models with various diameter of DSTJ and SD were investigated by the parameters, such as stress of neo-aortic root, change of the neo-aortic valve ring and neo-aortic valve cusps contact force during cardiac diastole.

## Methods

**Fig. 1:**
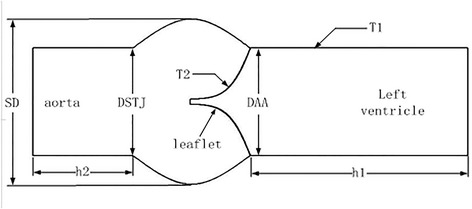
The geometric relationship of aortic root, including valvular leaflets, the valsalva sinus, ventricular outflow tract and the initial tract of the ascending aorta

Modeling neo-aortic root with ASO was accomplished by using a 3-dimensional (3D) tool of computer aided design. We created five neo-aortic valve geometric models with the different size (summarized in Table 1) of DSTJ and SD suggested by Labrosse [10–12], Haj-Ali [13] and Marino [14], namely model A, model B, model C, model D and model E (Fig. 1). Stress of neo-aortic root, diameter of neo-aortic valve ring and cusps contact force were simulated with a finite element model for structural mechanics.

**Table 1:** Geometric parameters in the 5 models, Unit: mm

Model	DSTJ	SD	hL	hS	h1	h2
A	9.70	12.30	8.84	13.02	5.00	5.00
B	11.60	12.30	8.84	13.02	5.00	5.00
C	7.76	12.30	8.84	13.02	5.00	5.00
D	9.70	14.76	8.84	13.02	5.00	5.00
E	9.70	9.84	8.84	13.02	5.00	5.00

### Geometry, mesh, tissue properties and boundary conditions of neo-aortic valve

**Fig. 2:**
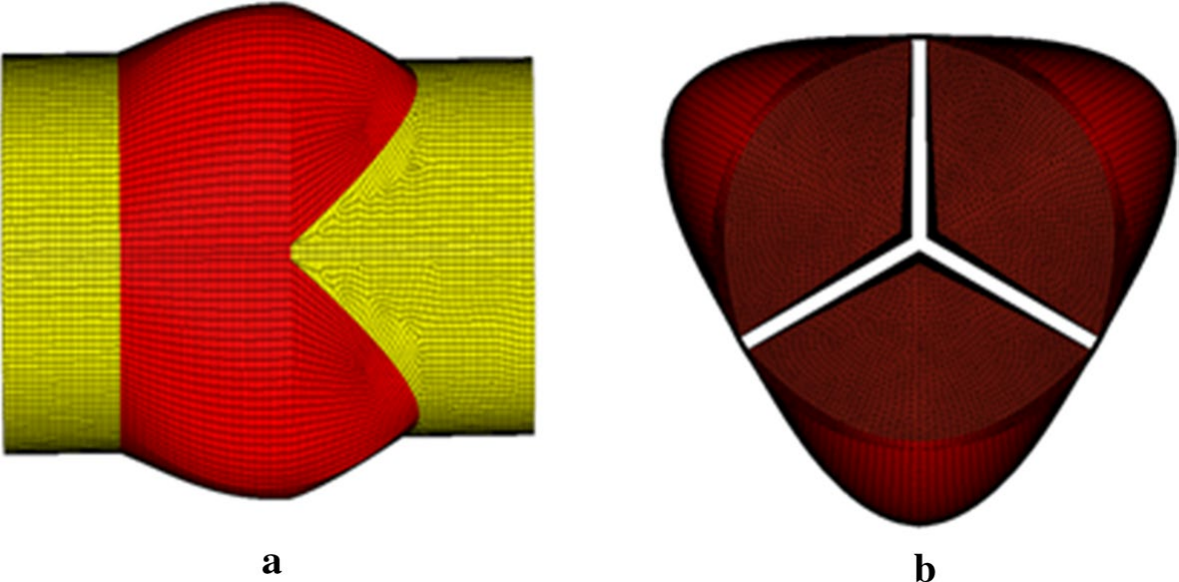
Finite element mesh of neo-aortic root. The geometries were meshed with shell elements. Three leaflets were meshed with triangular elements, and other parts of aortic root were meshed with quadrilateral element. **a** long axial view of reference model A; **b** short axial view of reference model A

The five 3D neo-aortic valves were created by SolidWorks (SolidWorks, Concord, MA). The parametric dimensions (DSTJ; SD; valve height, hL; sinus height, hS; h1, h2) were scaled with the size of neo-aortic valve ring (9.70 mm) [15]. A constant thickness of neo-aortic wall and the three cusps was 0.6 and 0.3 mm, respectively [13]. We took no account of twist and tilt of ascending aorta in geometric models. Rigid cylindrical parts (5 mm) on both sides of neo-aortic valve mimic the aorta and left ventricle duct, so as to apply the fixity and boundary conditions. The geometries were meshed with shell elements in HyperMesh (Altair Engineering, Troy MI). Three leaflets were meshed with triangular elements, and other parts of aortic root were meshed with quadrilateral element (Fig. 2). All models of neo-aortic valve steered automatic time stepping (ATS) manually. ATS can be used to vary the time step while no convergence is obtained in the original time step. The solver subdivides the time steps, and attempts to solve again. We conducted mesh-dependence trials with three sizes of mesh density (Table 2) for structural model of neo-aortic valve. Mesh2 and mesh3 increased the calculation steps than mesh1. So mesh1 has more element number but processes faster than mesh2 and mesh3. Mesh1 achieved satisfactory results in less solution time. We chose the mesh density which is the same as the mesh 1 and generated five meshes of neo-aortic valve (Table 3).

**Table 2:** Mesh independence analysis for structural mechanics simulations

Mesh model	Element number	Computation time (s)	Maximum stress (kPa)	Relative error
Mesh1	29,466	2649	96.29	–
Mesh2	24,138	3833	97.07	0.81%
Mesh3	24,484	3381	94.23	2.14%

**Table 3:** Number of elements and nodes

Model	Element number	Node number
A	29,466	24,117
B	32,921	27,195
C	31,979	21,721
D	36,372	25,206
E	35,960	24,802

We concentrated on closure performance of the neo-aortic valve during cardiac diastolic phase with normal diastolic blood pressure of 54 mmHg at six-month after birth [16, 17]. The valve model was then studied by applying known pressure load, as described by Zinner et al. [16]. The calculation models loaded with the peak pressure on the internal surface of neo-aortic, cusps surfaces and ventricular pressure to left ventricle inner wall [16]. In the neo-aortic valve models, the value of Young’s modulus and density were 1 and 2 MPa, 1100 and 2000 kg/m^3^ for cusps, ascending aorta and left ventricular duct [18, 19].

### Solution of the five neo-aortic valve models

The structural solver used a dynamics implicit method. To eliminate the numerical oscillation of the neo-aortic valve cusps, the Rayleigh damping factor β = 0.15 was adopted for all elements at every time step [18]. We adopted the constraint function algorithm to simulate the interaction among cusps of neo-aortic valve. Coulomb friction coefficient was 0.013 among the cusps. the five models of neo-aortic valves were simulated and post-processed by finite element code of ADINA 8.9 (ADINA R&D, Watertown, MA) used 4 cores on Xeon 8 3.60 GHz HP Z420 workstation with 16.0 GB RAM. Both the software version and computer are the same as our previous publication [20].

## Results

The closure performance of neo-aortic valve was investigated by the parameters, such as stress of neo-aortic root, variation of neo-aortic valve ring and cusps contact force during cardiac diastole.

### Stress of neo-aortic root

**Fig. 3:**
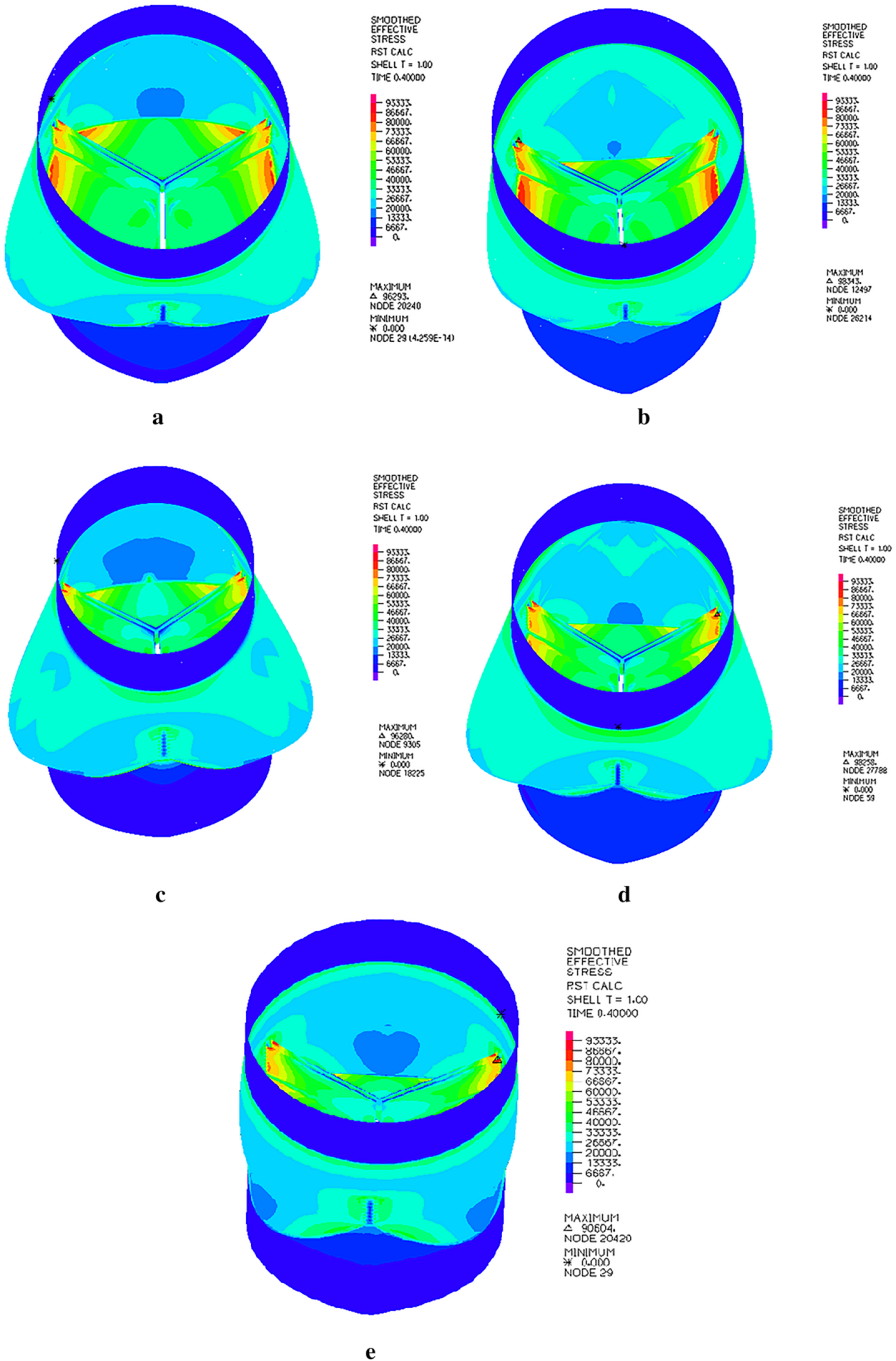
Stress of neo-aortic root during diastole for all models. The neo-aortic root models from **a** to **e** show the maximum stresses of 96.29, 98.34, 96.28, 98.26 and 90.60 kPa, respectively. Increasing the DSTJ and SD within a range of 20% can increase the maximum stress for neo-aortic root, and vice versa. **a** Model A: DSTJ = 9.70 mm, SD = 12.30 mm; **b** Model B: DSTJ = 11.60 mm, SD = 12.30 mm; **c** Model C: DSTJ = 7.76 mm, SD = 12.30 mm; **d** Model D: DSTJ = 9.70 mm, SD = 14.76 mm; **e** Model E DSTJ = 9.70 mm, SD = 9.84 mm

The approach described above successfully computed the closing phase of the neo-aortic root. The closure performance of neo-aortic valve was described from the calculated data. The quality of the closure can be seen from the maximum stress, because excessive stress values can damage the valve and reduce its durability [19]. The stresses of neo-aortic root in Fig. 3 depict that the highest stresses occur always at the top of commissures attachments. The locations of all structure model maximum stress agree well with simulated data by Labrosse [11]. The neo-aortic root model from A to E show the maximum stresses of 96.29, 98.34, 96.28, 98.26 and 90.60 kPa, respectively. Enlarging or narrowing the DSTJ and SD by 20% increase or decrease maximum stress for neo-aortic valve. Several research groups reported maximum stresses of healthy adult subjects in previous studies (range in 300–600 kPa) [10]. Comparing with the maximum stress of healthy adult subjects, the infant creates lower stress.

### Diameter of neo-aortic valve ring

We calculated the diameters of neo-aortic valve ring in the cardiac diastole period (Table 4). Diameters of neo-aortic valve ring were changed by 15.46, −24.74% enlarging or narrowing DSTJ by 20%. Diameters of neo-aortic valve ring were decreased by 14.43, 54.38% enlarging or narrowing SD by 20%. It is evident that increasing the DSTJ can decrease the diameter of the neo-aortic valve ring. Enlarging or narrowing SD can decrease the diameter of neo-aortic valve ring. Marom found that decreasing the aortic annulus diameter increased the coaptation height and area [19].

**Table 4:** Change of the aortic annulus diameter (CAAD)

Model	DSTJ (mm)	DS (mm)	CAAD (mm)	Relative difference
A	9.70	12.30	1.94E−02	0%
B	11.60	12.30	1.64E−02	−15.46%
C	7.76	12.30	2.42E−02	24.74%
D	9.70	14.76	1.66E−02	−14.43%
E	9.70	9.84	8.85E−03	−54.38%

### Contact force among neo-aortic valve cusps

We calculated the five neo-aortic valve models in the cardiac diastole to investigate closure performance with structural finite element method. Summation of nodes contact pressure was calculated to get the contact force among neo-aortic valve cusps, while enlarging or narrowing the DSTJ and SD. Contact force among neo-aortic valve cusps represents closure performance [19]. Contact forces (Table 5) among neo-aortic valve cusps are changed by 43.33, −10.00% enlarging or narrowing the DSTJ respectively by 20% compared. Contact forces among the neo-aortic valve cusps are changed by 6.67, −23.33% with SD varying 1.2× and 0.8× respectively. It is evident that enlarging and narrowing the DSTJ increase and decrease the contact force among the neo-aortic valve cusps respectively. Either enlarging or narrowing SD rise contact force among neo-aortic valve cusps.

**Table 5:** Contact force of aortic valve leaflet

Model	DSTJ (mm)	SD (mm)	Contact force (N)	Relative difference
A	9.70	12.30	0.30	0%
B	11.60	12.30	0.43	43.33%
C	7.76	12.30	0.27	−10.00%
D	9.70	14.76	0.32	6.67%
E	9.70	9.84	0.37	23.33%

## Discussions

Detailed working process of aortic valve has two phases. Several studies focused on the opening phase of the valve. Some metrics are used to evaluate the opening performance in terms of opening area, blood flow velocity, transvalvular pressure gradient, shear stress, maximum stress values [21, 22]. Several studies concentrated on cardiac diastole period. Some metrics are used to evaluate the closure performance, such as aortic valve cusps contact pressure, cusps coaptation and regurgitation [18, 19, 23, 24]. In this paper, we concentrated on closure performance of neo-aortic valve in the cardiac diastolic period.

Labrosse listed the dynamic analysis results in the literature, and showed that the maximum stress is within the range of 300–600 kPa which come from five research groups [18]. In the literature reported by Marom research group, the maximum stress is 350 kPa during the aortic valve closure [10]. In conclusion, the infant creates lower maximum stress than healthy adult subjects.

When the DSTJ and SD increase within a range of 20%, the increment leads to increasing the surface area of sinus inner wall and leaflet. If the aortic valve could close normally, it needs to generate more contact force among three leaflets. So enlarging or narrowing the DSTJ or SD will lead to neo-aortic valve regurgitation after a long period of time after the ASO to the patient with complete TGA. However, from hemodynamic perspective, in further studies, FSI method are necessary to simulate the parameters such as blood flow resistance, transvalvular pressure gradients, and energy loss, which are currently used for the hemodynamic evaluation of native heart valves. The parameters could increase with decreasing DSTJ and SD [25].

Previously we have investigated the effects of DSTJ and Maximum SD on Aortic Valve when the DSTJ of reference model A is 26 mm. It is evident that enlarging or narrowing the DSTJ and SD by 20% increases or decreases the neo-aortic valve cusps contact force respectively [26]. However, When the DSTJ of the reference model is 9.7 mm, it is evident that increasing or decreasing SD can decrease the change of the aortic annulus diameter and increase neo-aortic valve cusps contact force. As to the effect of different age groups on dynamic behavior of aortic root, some further considerations are necessary.

In this paper, we focused on the effects of geometric factors and ignored the effects of the material property on aortic root for the moment. For further study based on patient specific model, it is strongly needed to consider the effects of material property on calculation results. In physiological condition, the pressure load is non-uniform distribution on the leaflets and other part of neo-aortic root [27, 28]. Coronary orifices cause that pressure on the sinus inner wall drops in systole period of cardiac cycle. Additional studies should be performed with FSI method that could simulate the biomechanical performance of blood flow, aortic cusps and other parts simultaneously. So we could investigate closure performance with more metrics such as geometric orifice area, coaptation area, stroke volume, and regurgitation flow. Besides, we are trying to study on the aortic valve based on patient specific model. For example, we are studying surgical planning of aortic valve orifice direction for ASO. We are continuing to collect and analyze new cases with aortic valve disease before and after the operation. In further study, we will consider increasing both DSTJ and SD based on patient specific model. The structural finite element model descripted in this paper could use to investigate the closure performance and explore the stress, variation of neo-aortic valve ring and cusps contact force [29].

## Conclusion

We investigated the influence of varying the DSTJ and SD on the closure performance of neo-aortic valve after the ASO by structural finite element models. It is evident that enlarging or narrowing the DSTJ within a range of 20% can increase or decrease the maximum stress and the neo-aortic valve cusps contact force. Enlarging or narrowing the SD can decrease the change of the neo-aortic valve ring and increase the cusps contact force. It was a hint that varying the DSTJ and SD will lead to neo-aortic valve regurgitation after a long period of time after the ASO to the patient with complete TGA.
